# N-terminal pro-brain natriuretic peptide as a prognostic marker in patients with exacerbation of chronic obstructive pulmonary disease

**DOI:** 10.1186/cc12071

**Published:** 2013-03-19

**Authors:** P Stamatis, H Michalopoulou, D Stamatis

**Affiliations:** 1 Metaxa Hospital, Athens, Greece

## Introduction

Plasma N-terminal pro-brain natriuretic peptide (NT-pro-BNP) levels are elevated in patients with pulmonary disease especially in those with concomitant right ventricular dysfunction. The aim of the present study was to investigate the use of plasma NT-pro-BNP levels as a prognostic tool in patients with exacerbation of chronic obstructive pulmonary disease (COPD).

## Methods

A retrospective medical records analysis of all patients hospitalized between June 2009 and June 2012 with the final diagnosis of acute exacerbation of COPD who had undergone NT-pro-BNP measurements at admission followed by echocardiogram.

## Results

Seventy-two patients (mean age 69 years, 58% male) with COPD exacerbation but without clinical or echocardiography-oriented evidence of acute cardiac disease were enrolled. The mean forced expiratory volume in 1 second (FEV 1)/forced vital capacity ratio was 44.2% and the mean FEV 1 was 0.82 l. Median NT-pro-BNP levels at admission were 424.4 pg/ml and the tertile limits were 262.1 and 930 pg/ml. NT-pro-BNP levels significantly predicted 30-day mortality (OR: 7.2, 95% CI: 3.9 to 15.2; *P <*0.001), (OR: 25, 95% CI: 16.3 to 37.3; *P <*0.001) and (OR: 44, 95% CI: 44.5 to 76.2) for each tertile. These associations persisted after adjusting for arterial CO_2 _pressure, body mass index, age, gender and systolic pulmonary artery pressure.

## Conclusion

Among patients with acute exacerbation of chronic pulmonary disease, NT-pro-BNP could be a useful marker for severity and poor prognosis.

